# GObar: A Gene Ontology based analysis and visualization tool for gene sets

**DOI:** 10.1186/1471-2105-6-189

**Published:** 2005-07-25

**Authors:** Jason SM Lee, Gurpreet Katari, Ravi Sachidanandam

**Affiliations:** 1 Cold Spring Harbor Laboratory, Cold Spring Harbor, NY, USA

## Abstract

**Background:**

Microarray experiments, as well as other genomic analyses, often result in large gene sets containing up to several hundred genes. The biological significance of such sets of genes is, usually, not readily apparent.

Identification of the functions of the genes in the set can help highlight features of interest. The Gene Ontology Consortium [1] has annotated genes in several model organisms using a controlled vocabulary of terms and placed the terms on a Gene Ontology (**GO**), which comprises three disjoint hierarchies for *Molecular functions*, *Biological processes *and *Cellular locations*. The annotations can be used to identify functions that are enriched in the set, but this analysis can be misleading since the underlying distribution of genes among various functions is not uniform. For example, a large number of genes in a set might be kinases just because the genome contains many kinases.

**Results:**

We use the Gene Ontology hierarchy and the annotations to pick significant functions and pathways by comparing the distribution of functions in a given gene list against the distribution of all the genes in the genome, using the hypergeometric distribution to assign probabilities. **GObar **is a web-based visualizer that implements this algorithm.

The public website for GObar [2] can analyse gene lists from the yeast (S. cervisiae), fly (D. Melanogaster), mouse (M. musculus) and human (H. sapiens) genomes. It also allows visualization of the GO tree, as well as placement of a single gene on the GO hierarchy. We analyse a gene list from a genomic study of pre-mRNA splicing to demonstrate the utility of GObar.

**Conclusion:**

GObar is freely available as a web-based tool at [2] and can help analyze and visualize gene lists from genomic analyses.

## Background

Large scale genomic studies, especially expression microarrays, routinely identify large genes sets (sometimes a few hundred or more) of interest. Researchers are faced with the problem of identifying interesting features in such datasets. A listing of gene annotations (e.g. functions, process) can help identify interesting features, but this is impractical with large sets, due to the labor involved and the difficulty in picking statistically significant trends from large datasets. Thus, a user-friendly method is required for the routine analysis of such datasets.

The Gene Ontology consortium [1] curates the annotations of genes of several model organisms using a controlled vocabulary of GO terms. It also places the GO terms on a hierarchy called the Gene Ontology(**GO**). There are separate hierarchies for *Molecular Functions*, *Cellular Components *and *Biological Processes*. The terms on the hierarchy share a parent-child relationship in which a child term is either a specific instance or a part of its parent term. The terms get more specific the lower they are on the hierarchy [3]. Each node(GO term) on the hierarchy can have multiple parents and children.

**Figure 1:**
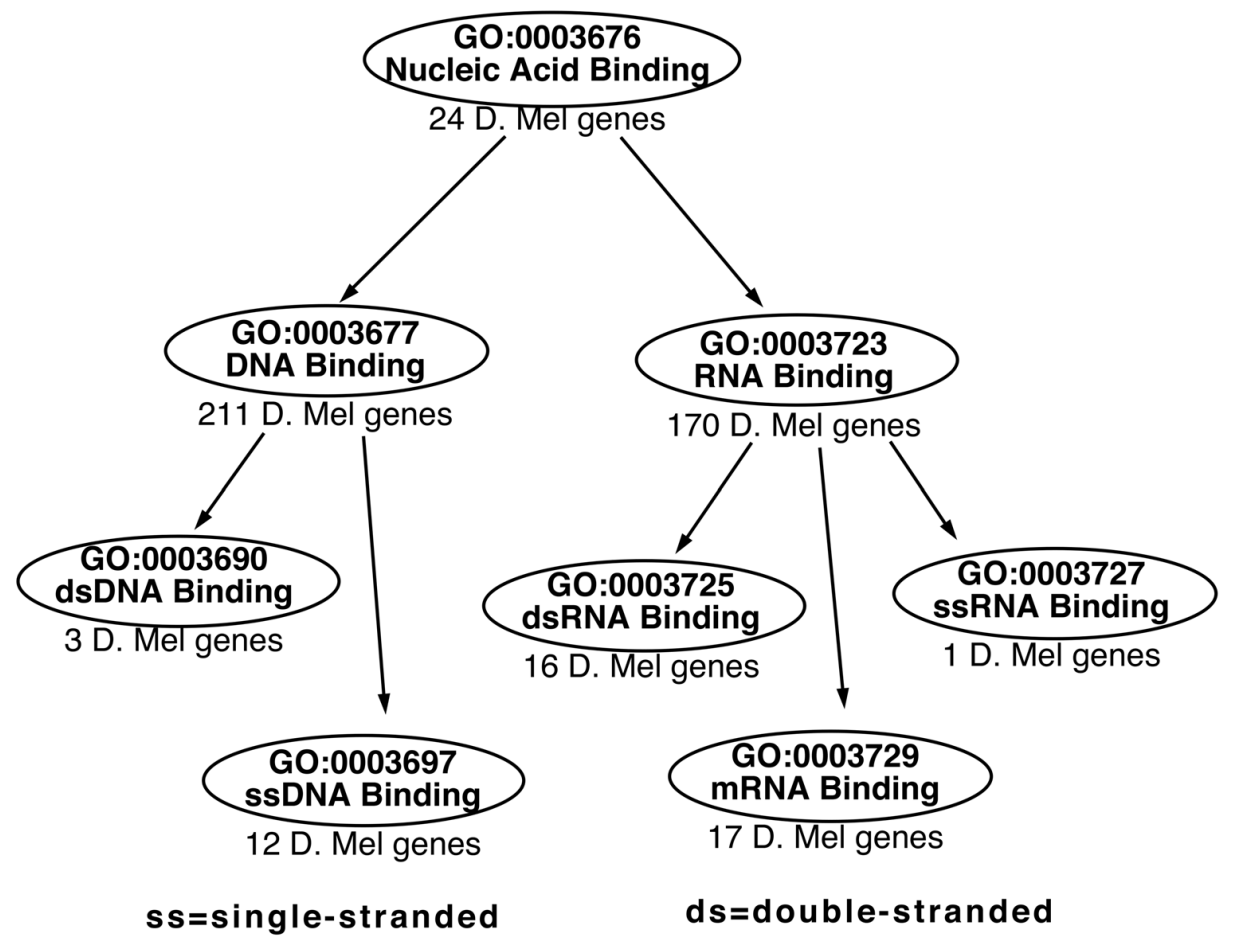
**A small section of the GO tree**. A schematic of a small section of the molecular function branch of the GO tree around the nucleic-acid binding term. The number of D. Melanogaster genes at each node is also given, as are the GO ids and the definitions of the terms at each node. The *GOTermBrowser *link at the GObar website [2] allows searching for GO terms using keywords and regular expressions (such as *NA*binding) and can also draw relationship diagrams as interactive images.

A small portion of the GO molecular function heirarchy around the *nucleic-acid binding *term is shown in Figure 1. This hierarchy can be generated using the *GO term browser *on the GObar website [2]. The GO term, GO:0003676, which corresponds to *nucleic-acid binding*, has two children, *RNA binding*(GO:0003723) and *DNA binding*(GO:0003677). RNA binding itself has many children, including *double-stranded RNA binding*(GO:0003725) and *single-stranded RNA binding*(GO:0003727). DNA binding has *double-stranded DNA binding*(GO:0003690) and *single-stranded DNA binding*(GO:0003697) as children.

**Figure 2:**
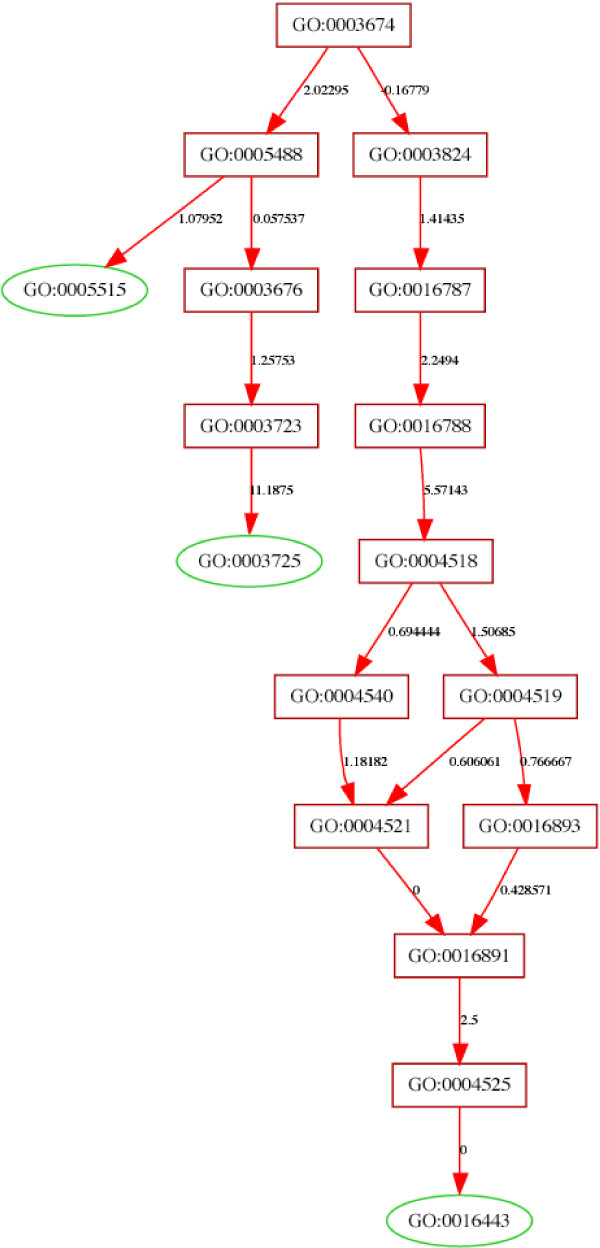
**Placement of D. Melanogaster Dicer-1 on the GO tree**. This is generated by entering FBgn0039016 (Dicer-1 in D. Melanogaster) on the Gobar website, and turning off pruning of the tree in step 4. The leaves (nodes with no children, here shown as green ovals) are the terms associated with Dicer-1. In GObar, green ovals signify nodes that contain genes from the uploaded list, while red nodes do not contain any genes from the uploaded list. In this case they correspond to amino-acid binding (GO:0005515), bidentate ribonuclease III activity (GO:0003725) and double-stranded RNA binding (GO:0016443). The numbers on the path, which signify deviation from the expected values, are used for pruning and highlighting highly interesting nodes, but are not important when pruning has been turned off.

A single gene can appear in several GO terms. For example, Dicer-1 (FBgn0039016 in D. Melanogaster) has several molecular functions, such as double-stranded RNA binding (GO:0003725) and bidentate ribonuclease III activity (GO:0016443) which are unrelated to each other. Figure 2 shows the placement of Dicer-1 on the GO tree.

In Figure 1, each node shows the number of genes annotated in the D. Melanogaster genome associated with the term. There are a total of approximately 14,000 genes in the genome. If a hypothetical microarray experiment in D. Melanogaster picks out 100 significant genes, of which 10 are double-stranded RNA (**dsRNA**) binding genes, then it is intuitively obvious that dsRNA binding is affected by the experiment and pathways using this function might be responding to the experiment. GObar uses the hypergeometric distribution (explained below) to quantify this intuition.

## Implementation

The basic idea of the algorithm is to compare the distribution of the genes from a set on the GO tree against the distribution of all the genes of the genome on the GO tree, identify and highlight branches that are improbably enriched by chance alone, and render an image of the GO tree that will allow user interactions to further explore the data.

The GO tree is first populated with all the genes in the genome, which involves placing genes at all the nodes that describe the gene. This operation is carried out only once, and is redone each time the genomic data gets updated. At each node two sets of counts are maintained, the contribution of genes from nodes that are children of the current node (distributed count) and the contribution from the genes at the current node (bare count). The total count at each node (bare count + distributed count), is divided equally amongst the distributed counts of each parent (please note that each node can have multiple parents). To calculate the counts at each node the leaves (nodes with no children) are first considered, and the counts are progressively transmitted up in a level-by-level manner, until the root is reached.

For the analysis the GO tree is populated with the list of genes to be analysed. Once again a set of distributed and bare counts is calculated at each node for this list. The distribution of these counts is compared to the genomic set and significance is assigned to the deviations from the expected counts, using the hypergeometric distribution, which is now described. If there is a collection of *N *objects of three types, *A *(count = *a*), *B *(count = *b*), and *C *(count = *c*) so that, *N *= *a *+ *b *+ *c*, then, a random selection of *n *objects from these *N *objects will contain *α A *objects, *β B *objects and *γ C *objects (where *α *+ *β *+ *γ *= *n*) with a probability given by the hypergeometric distribution



where,



This equation can be generalized to arbitrary collections of objects. Using this probability distribution, highly improbable deviations from the expected numbers are highlighted under the assumption that they are likely to have a biological significance.

## Methods

GO data can be downloaded from the Gene Ontology website [1]. The data contains two sets of information that are used, the parent-child relationships for each node and the definitions of each node or term. The data collection and analysis techniques are described in this section.

### Data acquisition

The downloaded GO data is used to populate one table with GO IDs and the ID definitions, and another table with a description of relationships between the GO IDs, which can use terms such as **is_a **or **part_of **to define the relationships.

In order to associate GO terms with gene IDs (accession), the files *gene2go *and *gene2accession *were retrieved from *Entrez Gene *[4] for the human and mouse genomes. A similar dataset for D. Melanogaster is acquired from *Flybase *[5]. Each gene can have multiple GO annotations, so this is a many-to-many association table.

A table, whose columns are shown in Table 1, is used to maintain node information, and to carry out statistical analysis. At each step of the methods listed below, one of the columns gets filled up. The columns in table 1 are filled in the following order,

**Table 1:** Columns in the GO statistics database table.

GO node statistics				
GOid	DC	BC	level	num of trails up

**Figure 5:**
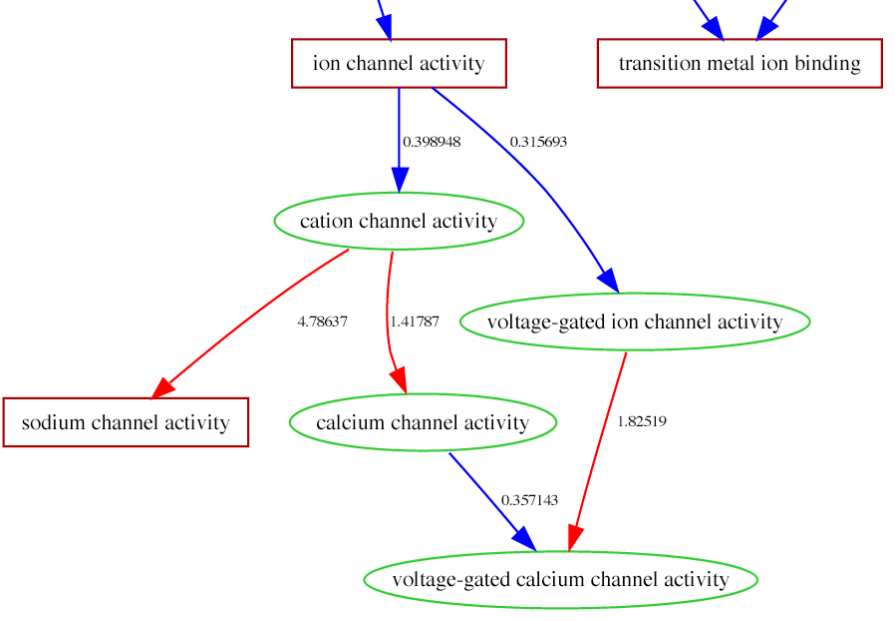
**Result of a GObar analysis of human genes with AT-AC-U12 type splice sites**. The result of a GObar analysis is an SVG (scalable vector graphics) image, with a red path signifying branches that are disproportionately over-represented in the gene list, as compared to the distribution of all the genes from the organism. Placing the mouse over a GO term pops-up a window in the figure, with information on the GO term and links to download data. The tool also allows searching for terms, as well as zooming in and out of the image. Table 2, which is a section of a table that appears in a pop-up window at the website at the end of the calculation, shows GO terms that are significantly enriched in the dataset. The numbers on each path depict the deviation over the expected count, this calculation is described in the text.

• Level (depth in a tree): A recursive depth-first search in a bottom-up fashion is carried out to determine the level of GO terms associated with the experiment, as explained in Figure 5.

• Number of trails up: This is obtained from the table of GO ID relationships by counting the number of parents for a node.

The following two items are calculated once in the beginning for all the genes in the genome and for each analysis of a gene-list.

• BC: Bare count (BC) is a number of genes associated with each GO term (node).

• DC: Starting from the lowest node(s) in the tree (determined by the Level column), the total count, *BC *+ *DC *is propagated to the node's immediate parent. If a node has more than one parent then total count is divided by the number of trails up, which is the same as the number of parents.

### Populating the tree with the reference dataset

We have populated the GO tree with datasets from *Entrez Gene *for human and mouse data [4], *Flybase *[5] for fly data and *SGD *[6] for yeast data. In the case of *Entrez Gene*, two sets of maps exist, a *gene id *to *GO *map and a *gene *to *gene id *map. At the end of this process each GO node gets a list of genes. The term *bare counts *denotes the counts of genes at each node. The genes on children nodes also contribute to the counts on any given node, which are tracked separately and called *distributed counts*. Thus, the distributed count of a node is the sum of contributions of the nodes below it in the gene ontology hierarchy. Each node contributes the sum of its bare count and its distributed count equally to the distributed counts of each of its parents. This process can be recursively applied, starting from the lowest levels (or greatest depths) of the tree and working the way up the tree.

If the accounting of distributed counts is to be done properly, defining the depth of each node in the tree is important. The rule for assigning depth to each node is that, if a node gets multiple levels, then the highest depth is always assigned to it. This can be done by picking the leaves of the tree (nodes with no children) and travelling recursively all the way up to the root (node with no parents). For each path to the top, depth is assigned to each node based on the number of steps to the node from the root. If a node already has a depth assigned to it, then the depth is replaced with the current depth only if it is bigger. This is explained in Figure 5. Once the leaves have been exhausted, all the nodes in the tree will have depths assigned to them.

In order to calculate the distributed counts for each node, the list of nodes is ordered based on their depths. Starting from nodes with the highest depths the counts are propagated up, as described above, summing up the bare count and distributed count and partitioning the sum equally amongst all the parents. After exhausting the list of nodes, all the nodes should have a bare count and a distributed count assigned to them.

### Populating the tree with the experimental dataset

In order to calculate probabilities for a given experimental dataset, we need to first populate the GO tree with the experimental dataset. A procedure identical to the one used in the previous section is implemented, resulting in a GO tree with just the dataset of interest on it.

### Calculating the probabilities

Let *BC_i_*, *DC_i _*be the bare and distributed counts respectively at node *i *for the genomic dataset and let *bc_i_*, *dc_i _*be the bare and distributed counts respectively at node *i *for the experimental dataset. Then, for the *Node 0 *in Figure 8.

*DC*_0 _= (*BC*_1 _+ *DC*_1_) + (*BC*_2 _+ *DC*_2_) + (*BC*_3 _+ *DC*_3_)/2     (3)

*dc*_0 _= (*bc*_1 _+ *dc*_1_) + (*bc*_2 _+ *dc*_2_) + (*bc*_3 _+ *dc*_3_)/2     (4)

The following are defined for ease of notation:

*N*_1 _= (*BC*_1 _+ *DC*_1_)     (5)

*N*_2 _= (*BC*_2 _+ *DC*_2_)     (6)

*N*_3 _= (*BC*_3 _+ *DC*_3_)/2     (7)

*N*_0 _= *DC*_0 _= *N*_1 _+ *N*_2 _+ *N*_3 _    (8)

*n*_1 _= (*bc*_1 _+ *dc*_1_)     (9)

*n*_2 _= (*bc*_2 _+ *dc*_2_)     (10)

*n*_3 _= (*bc*_3 _+ *dc*_3_)/2     (11)

*n*_0 _= *dc*_0 _= *n*_1 _+ *n*_2 _+ *n*_3 _    (12)

(13)

Then, the probability that a dataset is a random selection from the genes in the genome is given by the Hypergeometric formula (explained above)



where



The expected value for *n*_1 _is given by



We define *PD *as the deviation of the counts on a node *i *from its expected number and is given by



We use *P *and *PD *to prune the trees, as described in the next section.

### Pruning the tree

Listing all the nodes of the GO tree for a given dataset is not very informative, especially if only a few nodes are populated or if a large number of GO terms are populated by a small number of genes. This also defeats the purpose of helping users narrow down the GO terms of interest.

**Figure 3:**
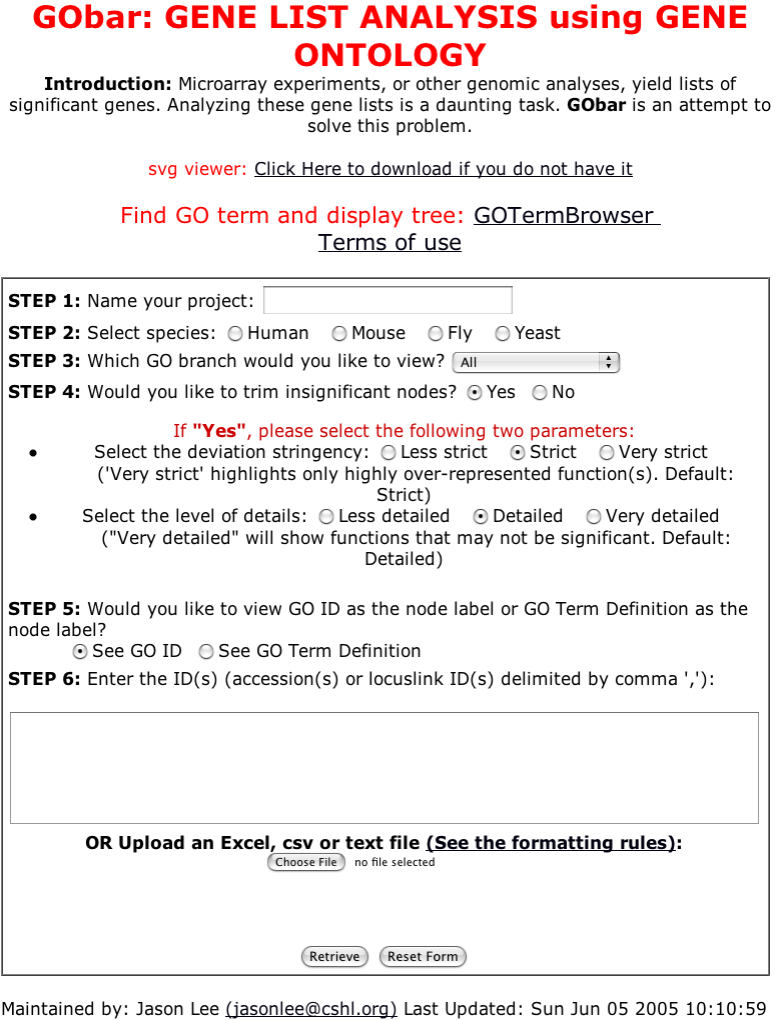
**The front page of the GObar website**. Selections are made for each step, and the list of genes is entered in the final step before launching the program. The pruning of the tree is controlled in step 4. A node can only be pruned if every node under it also satisfies the pruning condition. The pruning options are explained in the subsection **pruning the tree**. Very strict pruning might cause useful results to be thrown away, but can also highlight the best information in the dataset. In contrast, using a low stringency at this step, or no pruning, can cause too much information to be presented. Step 5 allows the nodes to be either annotated with GO ids (preferred for large trees) or definitions of GO terms. The *GOTermBrowser *link at the top of the page allows search for GOids using keywords or regular expressions such as "*NA*binding" to explore the GO tree neighborhood of the search term.

A node can only be pruned if every node under it also satisfies the pruning condition. The tree is pruned using the following rules to make the viewing manageable,

1. if *n*_0 _<*n_c_*, stop traversing the tree, that is, do not show anything below such a node. The population cutoff, *n_c _*can be set by the *level of details *option on the GObar webpage at step 4, shown in figure 3. This determines how low the population of genes in a node can go before it gets pruned. *Less Detailed *corresponds to a minimum of 6 genes, *Detailed *corresponds to a minimum of 3 genes and *Very Detailed *shows every node.

2. Prune nodes that have *P *> *P_th_*. The threshold *P_th _*is arbitrarily set at 0.1.

3. if *n_i _*deviates significantly up from <*n_i_*>, then the path is hightlighted using red color. *PD *can be set using the *deviation stringency *option in step 4 on the webpage, shown in figure 3. *Less strict *corresponds to a deviation cutoff value of 0.2, *Strict *corresponds to a cutoff of 0.5 and *very strict *corresponds to a cutoff value of 0.8.

The pruning is done starting with leaves (nodes with no children) on the tree, and stops when it reaches a node that should not be pruned according to the rules above. Fine variations of the pruning conditions are not allowed as these do not offer useful biological information and make the tool difficult to use.

### Visualizing the tree and user-interaction

Graphviz [7] is used to create the layout of the GO tree, and scalable vector graphics (**SVG**) is used to make it interactive. An example of the visualization is shown in Figure 2. Javascript is used to animate the SVG rendering of the GO tree. When the cursor is over a node, a window pops up with information on the GO term, genes that belong to the node and links to other resources. This window can be locked in place with a mouse click, allowing further exploration of the gene.

### Data download

The tool also allows the downloading of all the genes in the GO tree below any node. The downloaded list is in the form of a comma separated valued (csv) file, which contains the gene, the GO terms for each gene and a short description.

In the downloaded list, the uploaded genes are highlighted, since the list will also contain genes that belong to the nodes but are not in the uploaded list.

## Use of the tool

**Figure 4:**
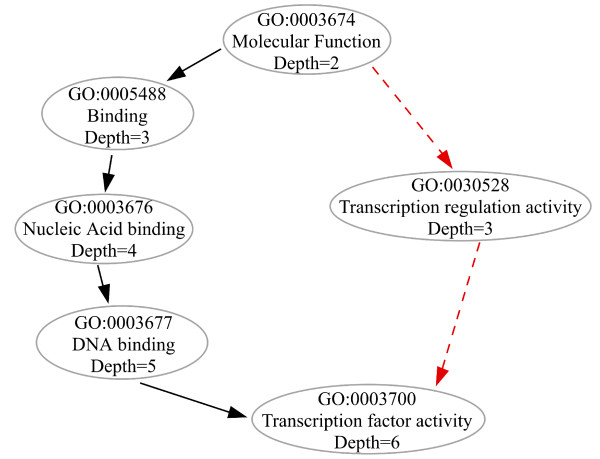
**GO tree depth calculation**. A section of the GO tree is depicted here. The directed acyclic nature of GO is shown by the red and black trails leading to the same node. The depth of a node is its distance from the root. Thus the node for GO:0003700 at the bottom has different depths on the tree, depending on the path traversed to get to it from the root. We use the higher number (6, the greater depth) as its depth for our calculations, which are described in the text.

Gobar is accessible at our website [2] and can analyze gene lists from the yeast (S. cerevisiae), fly (D. melanogaster), mouse (M. musculus) and human (H. sapiens) genomes. The front page of the website is shown in Figure 4. The list of genes to be studied can be entered into GObar by either uploading a file containing the list, or by entering the names in the text-area provided on the webpage. Each gene on the list can be annotated with user definitions, by using a colon to separate the gene name from the annotation (for example: *FBgn0034246:Dicer2, FBgn0039016:Dicer1*). The website offers several options to limit what is rendered in the result page, but using the default settings is recommended for the initial exploration. After the section of the GO tree relevant to the uploaded dataset is drawn, the website allows the user to limit the view to specific branches of the GO tree (either Molecular functions, Cellular components or Biological processes). Nodes containing genes from the uploaded list are rendered in a green oval. A node is red if it does not contain any genes from the uploaded list but one of its children node has genes from the list. A pop-up window also shows the GO terms and their levels in the GO heirarchy, that have been highlighted by the analysis. Taking the mouse over a GO term pops-up an information window that can by locked in place by clicking on the left mouse-button. There are links in this information window that can be used to download all the genes on and below the node on the GO tree, with the input genes highlighted in red. If user annotations are given for each gene, then they appear with the gene name in the data downloads. One of the links also allows listing all the GO terms below the node of interest on the tree. The genes in the data download that are not from the uploaded list might also be worthy of further study, especially if many of the other genes in the pathway or GO term are highlighted in the experiment.

The numbers on the lines between the nodes are the deviation from expected counts (whose calculation is described above). A red line implies that the child node connected to the path in the GO hierarchy is over-represented in the uploaded dataset and the corresponding GO term might be biologically significant.

If a single gene (or a few genes) needs to be placed on the GO tree, then the tree should not be pruned, which is achieved by selecting the "No" option in *step 4 *on the front page. Pruning is the removal of branches of the GO tree which do not carry much information (either a small number of genes or results that can be explained by random selection). The result of placing Dicer-1 (FBgn0039016) in D. Melanogaster is shown in Figure 2.

## Results

We will apply GObar to the analysis of results from a genomic study of splicing [8]. Splicing is the excision of introns from the pre-mRNA after transcription [9,10]. Broadly, there are two classes of splicing machinery (spliceosomes), U2-dependent and U12-dependent, named on the basis of the snRNPs involved in the splicing reaction. Each of these spliceosomes is involved in the excision of two sub-classes of introns, defined by the consensus sequences of the 5' and 3' ends of the intron, GT-AG-U2, GC-AG-U2, GT-AG-U12 and AT-AC-U12 type splice sites. The number of U12-dependent splice sites in the genome is dwarfed by the number of U2-dependent splice sites, numbering less than 2% of the total [8]. The snRNPs comprising the U12-dependent splicing machinery are also relatively less abundant. The D. Melanogaster, mouse and human genomes have U12-dependent splicing, while C. elegans seems to have lost it. This conservation leads to speculation regarding the reason for the persistence of the U12-dependent splice sites over evolutionary time scales.

If the U12-dependent splice sites persist for some biological reason, then it seems reasonable to assume that only genes with roles in certain functions should contain these splice sites. There has been some speculation, but no rigorous assessment, regarding the functional bias of these genes [9,10]. A comprehensive annotated collection of splice sites for the worm (C. elegans), fly (D. melanogaster), mouse (M. musculus) and human (H. sapiens) genomes has been generated by classifying the known sites with the help of human curation [8]. The list of genes with AT-AC-U12 type splice sites was analyzed using GObar to identify functional themes that might be highlighted. Figure 5 shows a part of the result of GObar analysis of the human genes with AT-AC-U12 type splice sites. Table 2 shows the GO terms that are highlighted by the analysis of the human set.

**Table 2:** Significant GO terms in the human AT-AC-U12 set, which accompanies the analysis shown in Figure 5. We show only the more specific terms in the table that appears as a pop-up webpage along with the GO tree shown in Figure 5. The level is the depth from the root, and its calculation is described in Figure 4.

Cellular component			
GOid	Function	Type	Level

GO:0030176	integral to endoplasmic reticulum membrane	component	7
GO:0001518	voltage-gated sodium channel complex	component	6
GO:0005891	voltage-gated calcium channel complex	component	6
GO:0008023	transcription elongation factor complex	component	6
GO:0005667	transcription factor complex	component	6

Molecular Function			

GO:0008332	low voltage-gated calcium channel activity	function	9
GO:0005248	voltage-gated sodium channel activity	function	8
GO:0005245	voltage-gated calcium channel activity	function	8
GO:0005262	calcium channel activity	function	7
GO:0005272	sodium channel activity	function	7

Biological Process			

GO:0006814	sodium ion transport	process	9
GO:0006816	calcium ion transport	process	9
GO:0030029	actin filament-based process	process	9

In the molecular function branch, analysis of the mouse set highlighted *GTPase regulator activity*, *cation channel activity *and *calcium channel activity*. The human set also highlighted *sodium channel activity *and *voltage-gated calcium channel activity*. In the biological process branch, the analysis highlighted *intracellular signalling cascade *and *actin-filament based process*. The Drosophila set was too small (7 genes), to give any detailed statistics, but GObar did highlight *transporter activity*, which is a parent of the *cation channel activity*. The highlights that are present in both human and mouse genomes could point to reasons for the persistence of AT-AC U12-dependent splice sites over evolutionary time-scales. We believe this might have some basis in the biological control of the rates of splicing reactions of these genes but reaching a firm conclusion requires an investigation that is beyond the scope of this study.

## Discussion

Our proposed method of analysis is mathematically robust and allows visualization and identification of pathways. We can identify sub-groups of genes that cannot be explained by chance alone. This in turn can identify pathways that are of interest in the process under study. Identification of the pathways then allows study of other genes in the pathway that are not picked up in the experiment, allowing for a clearer understanding of subtle effects and quantifying the errors in the experiment.

The conclusions we reach using our method of analysis does depend on the accuracy of the gene annotations. Thus, if the role of a gene in a pathway were unknown, or if a small set of genes could have a strong phenotypic effect (without triggering major changes in the mRNA levels of a large number of genes, which is the only quantity measured in a microarray experiment), then GObar will mislead the investigator. Such phenomena are, in general, more difficult to study using microarrays and require supplementary biological assays to uncover the underlying mechanisms.

Using the GO tree allows us to ameliorate some of the problems inherent in gene annotations, so that genes below the term of interest are also counted as part of the function being studied. Our method involves a one-time analysis of the whole genome dataset, which then allows us to decide, in a straightforward manner, the significance of any number of datasets and allows easy navigation and analysis of the data. The convenience and robustness of the method are the novel contributions here.

Another rigorous approach identifies biological themes from gene lists using GO, by calculating the over-representation of categories in the experimental list relative to a background list (all genes on the chip or all genes in the genome). A problem with this is the underlying assumption that the GO annotations at each node are accurate. In our approach, in order to allow for the fact that genes may be placed at different depths due to human biases, we let every gene below a particular node contribute to the counts on that node, but done in a way that prevents multiple counting. This also allows genes with more specific functional annotations to contribute to the more general annotation. Thus, our approach also differs from this one in the way we define *hits*, by allowing genes that are lower down in the tree to be a part of the node under consideration.

We offer a detailed comparison with GOstat [11] available at its website [12]. GOstat returns lists of significantly enriched GO terms and genes in those GO terms. In order to see the meaning of the term and its placement in the GO tree the user can click on the link to go to AMIGO [13]. The results are comparable, though we do not offer a P value for the significant nodes. They also offer a list of GO annotations for each gene in the uploaded list. A list of terms is not human friendly and is not a natural method of presenting data that has a tree-like structure. We feel that our SVG based view of the tree is intuitively easier to use and also allows for a quick overview of the data, allowing the user to zoom into relevant sections. Also, our listing of the functions in order of their level in the GO tree allows a user to pay attention to more nodes based on levels (more general corresponds to lower level, while higher levels correspond to more specific terms). The goal of the study determines what level of specificity for a node is preferred.

In addition, the GO browser offered on the GObar website allows an SVG-based exploration of the GO tree, simultaneously showing all the branches and relationships between them, which is different from the text based version offered by the AMIGO website [13]. GObar also allows viewing all the GO annotations of a gene in a single view, as shown in figure 2, which is also a novel feature of this program.

The analysis of genes with U12-dependent splice sites, given in the previous section, is indicative of the power of this approach. We identified functions that are over-represented in the AT-AC-U12 set, which in turn can be the starting point of an investigation into the phylogeny of the genes involved. The phylogeny could explain the evolution and the role of AT-AC-U12 type splice sites.

## Conclusion

GObar is a convenient tool for the analysis of large gene lists. It provides useful guidance to biologists on functions and pathways that need further study and is available freely over the web at [2].

## Authors' contributions

Ravi Sachidanandam conceived the algorithm and the project and helped with the coding. Gurpreet Katari implemented an initial version of the code before leaving his position and Jason Lee improved and revised much of the code and implemented the web-based front end.

**Figure 6:**
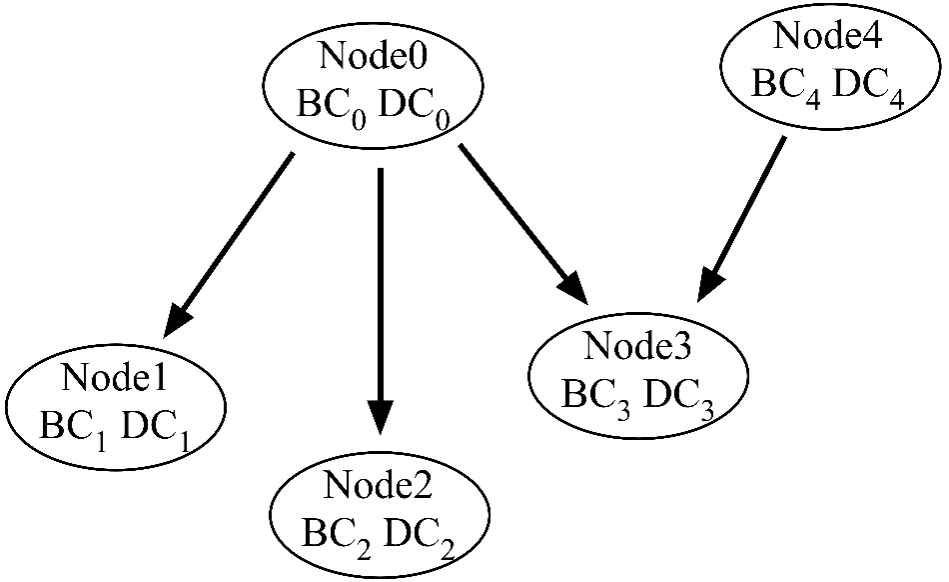
**Calculation of the bare and distributed counts of genes at each GO term**. The nodes in the figure are GO terms, the arrows are directed from parent to child nodes. The bare count (BC) at each node is the number of genes that are placed there by the annotations of the gene lists. The distributed counts (DC) are the counts transmitted up from the children of the node. Each node contributes its *total count = bare count + distributed count*, equally up to each of its parents. Thus half of the total number of genes in *Node 3 *are contributed to the distributed counts of *Node 0 *and *Node 4*.
